# Alternative Fish Species for Nutritional Management of Children with Fish-FPIES—A Clinical Approach

**DOI:** 10.3390/nu14010019

**Published:** 2021-12-22

**Authors:** Gavriela Feketea, Emilia Vassilopoulou, Foteini Geropanta, Elena Camelia Berghea, Ioana Corina Bocsan

**Affiliations:** 1 PhD School, Iuliu Hatieganu University of Medicine and Pharmacy, 400337 Cluj-Napoca, Romania; gabychri@otenet.gr; 2 Department of Pediatrics, Amaliada Hospital, 27200 Amaliada, Greece; 3 Department of Pediatrics, Karamandaneio Children’s Hospital, 26331 Patras, Greece; 4 Department of Nutritional Sciences and Dietetics, International Hellenic University, 57400 Thessaloniki, Greece; vassilopoulouemilia@gmail.com; 5 Allergy Private Office Galatsiou, 11147 Athens, Greece; fgeropanta@gmail.com; 6 Department of Pediatrics, Carol Davila University of Medicine and Pharmacy, 020021 Bucharest, Romania; 7 Department of Pediatrics, “Marie S. Curie” Emergency Children’s Clinical Hospital, 041451 Bucharest, Romania; 8 Department of Pharmacology, Toxicology and Clinical Pharmacology, Iuliu Haţieganu University of Medicine and Pharmacy, 400337 Cluj-Napoca, Romania; bocsan.corina@umfcluj.ro

**Keywords:** food protein-induced enterocolitis syndrome (FPIES), fish, bony fish, cartilaginous fish

## Abstract

In the Mediterranean region, fish is a common cause of food protein-induced enterocolitis syndrome (FPIES) in children. No laboratory tests specific to FPIES are available, and oral food challenge (OFC) is the gold standard for its diagnosis and testing for achievement of tolerance. Children with FPIES to fish are usually advised to avoid all fish, regardless of the species. Fish are typically classified into bony and cartilaginous, which are phylogenetically distant species and therefore contain less cross-reacting allergens. The protein β-parvalbumin, considered a pan-allergenic, is found in bony fish, while the non-allergenic α-parvalbumin is commonly found in cartilaginous fish. Based on this difference, as a first step in the therapeutic process of children with FPIES caused by a certain fish in the bony fish category (i.e., hake, cod, perch, sardine, gilthead sea bream, red mullet, sole, megrim, sea bass, anchovy, tuna, swordfish, trout, etc.), an OFC to an alternative from the category of cartilaginous fish is suggested (i.e., blue shark, tope shark, dogfish, monkfish, skate, and ray) and vice versa. Regarding the increased mercury content in some sharks and other large species, the maximum limit imposed by the European Food Safety Authority (EFSA) for weekly mercury intake must be considered. An algorithm for the management of fish-FPIES, including alternative fish species, is proposed.

## 1. Introduction

Food protein-induced enterocolitis syndrome (FPIES), a non-IgE-mediated food allergic disorder, can be induced by a wide range of foods. The rate of fish as the offending food in children with FPIES varies among geographic locations. Many studies have shown that fish is among the frequent causes of FPIES in the Mediterranean basin, where fish is a common dietary constituent [1,2,3,4,5,6,7,8,9,10,11]. In addition, in adults and adolescents, FPIES may be provoked most commonly by seafoods, including fish [12,13,14,15]. Observational studies have shown that a certain percentage of people with FPIES caused by one species of fish may tolerate other species [16]. Here, we discuss from the allergist’s point of view the different species of fish used for human consumption and review the current evidence on tolerance across fish species, with suggestions on which species would be the most suitable for conducting the first oral food challenge (OFC) in children with FPIES caused by fish.

## 2. Fish in the Human Diet

Fish is a widely available food, which is highly nutritious due to its rich content of high-quality proteins and polyunsaturated fatty acids (PUFAs). Studies have shown that the regular consumption of fish can enhance health and quality of life (QoL) in various ways, improving vision in childhood and reducing the risk of cardiovascular disease (CVD) [17]. In recent years, there has been a general tendency toward the adoption of a healthier diet, which has led to an increase in fish consumption. According to the Food and Agriculture Organization Corporate Statistical Database (FAOSTAT), sea fish consumption in Europe in 2017 was 24.35 kg/citizen/year [18].

The fish market differs among countries, depending on availability, dietary habits, and economic status. The most commonly consumed fish worldwide are *Gadiformes* (cod, hake), *Salmoniformes* (trout, salmon), *Cypriniformes* (carp), *Clupeiformes* (sardine), *Siluriformes* (catfish), and *Poerciformes* (tuna, mackerel) [19,20]. Carp is among the most popular fish in Asia, while salmon and cod are preferred in northern Europe. Families in Japan and the United States (US) prefer salmon, tuna, and mackerel [21,22]. In terms of preparation, fish can be eaten uncooked, marinated, smoked, fried, grilled, steamed, roasted in the oven, boiled, or baked, according to local customs and individual or familial preferences, and some species are canned. Fish species may differ in their allergenic potency, which is not the same as that of shellfish (e.g., crustaceans, mollusks) [23].

### 2.1. Classification of Fish

Currently, more than 30,000 species of fish are recognized. According to their biological characteristics, they are divided in two classes: *Osteichthyes* (bony) and *Chondrichthyes* (cartilaginous). Cartilaginous fish account for about 7% of the global biodiversity, and they are further divided in two subcategories, the *Elasmobranchs* (sharks, rays, skates, and sawfish) and the *Holocephali* (chimaeras) [24]. Bony fish or teleost fish consist of 45 orders and more than 430 families; they are divided into *Actinopterygii* and *Sarcopterygii* based on their fin shape [25]. Only a limited number of orders are consumed by humans, specifically, the salmon-like (*Salmoniformes*), cod-like (*Gadiformes*), perch-like (*Perciformes*), herring-like (*Clupeiformes*), carp-like (*Cypriniformes*), catfish-like (*Siluriformes*), and flatfish (*Pleuronectiformes*) [23].

The most obvious difference between bony and cartilaginous fish is in their endoskeleton. The bony fish skeleton is composed exclusively of bones, while that of cartilaginous fish is composed of cartilage. Teleost fish are the largest infraclass in the class *Actinopterygii* (ray finned fish) [26]. They have two types of muscle, which are both used for swimming. The light (white) muscle is used for short bursts and the red (dark) is used for constant swimming [27]. In contrast, the cartilaginous exoskeleton consists of small denticles coated with sharp enamel [24].

The gadiform fish (order *Gadiformes*) belong to the bony fish category, comprising 8 families, 59 genera, and more than 180 species, and they contribute more than one-quarter of the world’s marine fish catch [20]. Bony fish that more frequently induce FPIES are cod (*Gadus morhua*), hake (*Merluccius merluccius*), perch (*Perca fluviatilis*), sardine (*Sardina pilchardus*), gilthead sea bream (*Sparus aurata*), red mullet (*Mullus barbatus*), sole (*Solea solea*), megrim (*Lepidorhombus whiffiagonis*), sea bass (*Dicentrarchus labrax*), anchovy (*Anchoa*), tuna (*Thunnus*), swordfish (*Xiphias gladius*), and trout (*Salmo trutta*) [2,3,4,5,6,9,10].

Blue shark (*Prionace glauca*), dogfish (*Scyliorhinus canicular*), monkfish (angelshark—*Squatina squatina*), skates and rays (*Raja*), and tope shark (*Galeorhinus galeus*) are cartilaginous fish that are part of the human diet [28]. In view of the high mercury content of large ocean fish, such as swordfish, sharks, and fresh tuna, several regulatory bodies recommend avoiding the consumption of these fish by pregnant women and young children [29,30]. In this regard, the European Food Safety Authority (EFSA) established a tolerable weekly intake (TWI) for methylmercury of 1.3 µg/kg body weight (bw) and of 4 µg/kg bw. for inorganic mercury [31,32]. However, certain species of shark, including the blue shark, which focus their foraging behavior on prey of the mesopelagic zone, have a lower mercury content [33]. Taylor et al. showed that for all species, mercury content was directly related to size and age; larger, older specimens had higher concentrations of mercury than smaller, younger sharks and skates [34]. The US Food and Drug Administration (FDA) recommend skate as one of the best choices of fish to be consumed during pregnancy, breastfeeding, and early childhood [29]. As exposure to methylmercury above the TWI is of concern, supervision of children’s diet by a specialist and/or dietician is recommended.

### 2.2. Fish Allergy and Fish Allergens

Food allergy affects around 5% of adults and 8% of children [35]. It is an adverse reaction to foods or food additives, and it can be differentiated into allergic hypersensitivity, which involves an immune mechanism and non-allergic hypersensitivity. It is further differentiated into IgE-mediated and non-IgE mediated [36]. Fish allergy presents in 0.2% to 2.29% of the general population, varying according to regional dietary habits, fish species exposure, and ways of preparation and cooking [19,37]. In children, only a few FPIES caused by both fish and shellfish (crustacean and cephalopods) are reported [3]. In crustacean and cephalopods, the major allergen responsible for ingestion-related allergic reactions is the muscle protein, tropomyosin [37]. Parvalbumins, enolases, and aldolases are present in fish muscles, the first being the major allergen [38,39]. Different fish species have been shown to exhibit various degrees of parvalbumin allergenicity.

### 2.3. Parvalbumins

There are two subtypes of parvalbumins, according to their phylogenic origin, the α-protein lineage and the β-protein lineage. The β-parvalbumin subtype is encountered in fish and is responsible for almost 95% of IgE-mediated hypersensitivity to fish [38]. The parvalbumins are calcium-binding sarcoplasmic muscle proteins, with a molecular weight of 12 kDa (108–109 amino acids). Fish parvalbumins are highly water-soluble and exhibit resistance to heat, denaturing agents, and extreme pH. They consist of three EF-hand motifs with two high-affinity calcium ion-binding sites [23]. Their ability to buffer calcium (Ca) plays a role in muscle relaxation, and their allergenic potential is significantly reduced when Ca is removed. Ion binding is a key to the parvalbumin stability, and parvalbumins lacking Ca^2+^ bind only weakly to IgE antibodies from fish-allergic patients [38,40]. The parvalbumin found in cartilaginous fish (α-parvalbumin), is characterized as non-allergenic, while the parvalbumin of bony fish (β-parvalbumin) is considered pan-allergenic [27,38]

Fish allergens have been investigated in nearly 40 species, although most European studies have concentrated on common local fish, such as cod, salmon, carp, and tuna. Fish muscle exhibits the greatest allergenicity, and parvalbumin and to a lesser extent enolases and aldolases are the major allergens in fish muscle [39]. Studies have shown that there is variation in the allergenicity of parvalbumin between different fish species [20,27,41,42,43,44,45,46,47]. Parvalbumin has been most extensively studied in the following bony fish: Atlantic cod (*Gadus Morhua*), Alaska pollack (*Theragra Chalcogramma*), common carp (*Cyprinus Carpio)*, silver carp (*Hypophthalmichthy Molitrix*), Atlantic salmon (*Salmo salar*), and more recently Asian seabass (*Lates calcarifer*). Regarding cartilaginous fish, the role of parvalbumin has been investigated in blue shark (*Prionance glauca*), salmon shark (*Lamna ditropis*), spotless smooth-hounds (*Mustelus griseus*), and halibut (*Hippoglossus stenolepis*) [47]. There is little evidence on coexisting allergy to α- and β-parvalbumin, which suggests low clinical cross-reactivity between them [38]. The main differences between the lineages are the presence of more acidic amino acid residues in β-parvalbumin and differences in length (≥109 amino acids in α-parvalbumin compared with <109 in β-parvalbumin) [38]. *Chondrichthyes* express low levels of allergenic fast muscle. The variation in the parvalbumin amino acid sequence between different fish species and lineages appears to play an important role in patient sensitization. The difference in amino acid sequence between different parvalbumins could be an indication of the likelihood of clinical cross-reactivity [20].

In a Japanese study, the investigators heated the flesh of seven bony fish (mackerel, red seabream, yellowfin tuna, silver salmon, Japanese sardine, chicken grunt, goldeye rockfish) and four cartilaginous fish (bigeye thresher, shortfin mako shark, mottled skate, blue shark) to different temperatures and for different times, with the aim of determining the thermostability of fish collagen as an allergen. They found that cartilaginous fish produced less IgE reactivity than bony fish and suggested that the allergenicity of cartilaginous fish collagen is lower than that of bony fish [48]. Kobayashi et al. observed that in bony fish, regardless of fish species, there is less parvalbumin in the dark than in the white muscle. They concluded that the more commonly consumed fish with white muscle is more likely to be allergenic [27]. In tuna, parvalbumin was found in the white muscle but not in the red, and it was also unequally distributed in different parts of the muscle [49]. Another study suggested that the method used to prepare the fish, and the duration of heating, can affect the parvalbumin epitopes, leading to alterations in the allergenicity [46]. Children affected by IgE-mediated fish allergy appear to show a higher tolerance for canned tuna [50]. While heating caused a reduction in antibody reactivity to multimeric forms of parvalbumins in most bony fish, a complete loss of reactivity was observed for cartilaginous fish [46,51]. This is another reason to select cartilaginous fish for the first OFC in a FPIES. Apart from the phylogenetic differences of the allergens, the heating process contributes to the reduced allergenicity of cartilaginous fish. Based on these findings, regardless of the species chosen, we recommend that OFC is conducted with fish baked in the oven at 160 °C for at least 30 min.

## 3. Food Protein-Induced Enterocolitis Syndrome (FPIES) Caused by Fish

The culprit food differs according to the age of onset of FPIES and depends on the time when the culprit food is introduced in a baby’s diet, although cases of adult onset have also been reported [12,13,14,15,52,53,54]. FPIES caused by milk and soy usually develops in the first 3 months of life, while FPIES caused by grains presents between the 5th and 7th month, as grains are more likely to be introduced into the infant’s diet at this stage [55]. Fish is usually introduced to children’s diet after the 6th month and in certain regions after 12th month, and thus, a delay in FPIES caused by fish is observed [56,57], and children persistently reactive to fish were reported to be diagnosed at a significantly older age than those reactive to milk [9].

From a geographically diverse population of 441 children with FPIES, data provided by caregivers in the International FPIES Association showed that fish was the third lowest in the hierarchy of offending foods [58]. In contrast, reports from Mediterranean countries, specifically Greece [8,9,10], Italy [2,3], Spain [1,4,5,6,7,16,59], and Turkey [60] revealed fish as the first or second most commonly implicated food and a major trigger of solid food protein allergy. It has also been concluded that the resolution of fish-FPIES comes later than that from other foods [61], and many children with fish-FPIES will not overcome the disease during childhood [62]. The current guidelines recommend periodic re-evaluation with supervised OFCs to monitor for resolution [55,57]. Results from studies of children with IgE-mediated fish allergy suggest that they may be able to tolerate fish species other than those associated with the initial onset of symptoms [63]. Observational studies of fish-FPIES show that a certain percentage of children tolerate species other than fish identified as the culprit [16]. As no laboratory analysis or dermatological test is available to predict when tolerance to the offending or alternative fish has been achieved, all children with fish-FPIES should undergo a periodic OFC. Several protocols for OFC in fish-FPIES have been proposed [2,4,5,64,65], and optimal challenge procedures can be unclear to practitioners and underutilized [66]. The suggested OFCs vary regarding the amount of protein/food served per dose, the time between doses, and the duration (one day, or more non-consecutive days). The current consensus and guidelines do not clearly specify whether the OFC should be conducted with the offending fish or with an alternative species, and if the latter, which species [61]. Infante et al. showed that the probability of not presenting a reaction during OFC was four times higher in children with FPIES who received an alternative fish than in children who received the culprit fish; of 32 patients tested to an alternative fish, 27 had a negative OFC [16], and the researchers proposed to challenge first with an alternative fish [67]. In addition to bony fish, blue and tope sharks, skates, and rays are also commonly used in the Mediterranean cuisine. In our experience, children with both IgE and non-IgE-mediated reactions to hake, cod, or other culprit fish have tolerated cartilaginous fish earlier.

## 4. The Clinical Approach to Investigation of Tolerance across Fish Species

The observation of differences in allergenicity among fish species has directed researchers toward exploring the tolerance of patients to fish other than the culprit species, with the aim of establishing an alternative option and avoiding a restricted diet [3,10,11,16,68,69].

The various pediatric societies have issued no specific instructions on which fish species should be introduced first to the diet of infants/children. In several countries, hake (*Merluccius merluccius*) is commonly recommended by pediatricians as the fish to be tried first, even before the age of 1 year. It is one of the fish most frequently consumed in Mediterranean countries, but it is also one of the fish most commonly specified as the offending fish in cases of FPIES. It can be found fresh throughout the year, is a small fish, is easy to prepare, and is not very expensive. In addition to its light smell and taste, which makes it more acceptable to children, hake has fewer small bones, so the danger of these being swallowed by babies and children is low.

Most studies do not specify which species of fish is the FPIES culprit under investigation; instead, they refer to it as ‘fish’, ‘cod’, or ‘codfish’ [2,8,70,71,72]. Data mainly from the Mediterranean countries refer to hake as the most commonly offending fish [1,4,5,6,7,11,16]. *Merluccius merluccius* is also one of the most frequently involved fish species in adult-onset FPIES [15]. Hake (*Merluccius merluccius*) belongs to the *Merlucciidae*, a family of cod-like fish of the genus *Merluccius.* Hake and cod are both white-fleshed fish belonging to the *Gadiformes* order. The Atlantic cod belongs to the *Gadidae* family of the genus *Gadus* in the *Actinopterygii* class. Many fish throughout the world that have the word “cod” in their name do not belong to the genus *Gadus* [41]. In Europe, the most commonly consumed bony fish are Gadiforms, such as cod and hake, and the most commonly consumed cartilaginous fish are sharks and rays [19]. Geographical differences are documented in the prevalence of fish allergy and the type of fish causing allergy, possibly due to cultural and dietary differences, and differences in the distribution of fish. In Asia, the most frequently reported causative agents are anchovy and mackerel, while in South Africa, hake (24.8%), yellowtail (32.9%), salmon (15.2%), and mackerel (15.2%) are the most common culprits [19]. In Europe, in children with FPIES, cod and hake are the more frequent offenders. Table 1 shows the current evidence on the distribution of fish found responsible for FPIES in children and the tolerance to other species.

Some studies were able to demonstrate that patients with FPIES caused by certain types of fish could tolerate other fish species [16,67], although Sopo et al. reported that two patients presented with late FPIES symptoms due to the alternative fish, despite having tolerated it well previously [78]. This happened with sole and tuna, respectively, in two patients with initial FPIES to hake and cod, and sole and cod, respectively. It should not be assumed that there will be tolerance to a particular fish when a fish belonging to the same order is well tolerated. The mechanisms behind differential tolerance to fish of the same order are not well understood. It has been suggested that there could be an inappropriate adaptive immune response to the protein component of foods, similar to that encountered in IgE-mediated allergy. In addition, it is not clear which fish allergen triggers the FPIES. In the cases described by Sopo et al., patients suffering from FPIES to one fish had already experienced several uneventful ingestions of the alternative fish prior to the onset of symptoms. It is conceivable that the allergen of the alternate fish is similar to but not exactly the same as that of the offending fish, and thus, it appears initially to be tolerated, but subsequently, the immune system recognizes it as foreign, triggering the allergic reaction. Another possible explanation is that due to the loss of immunological memory, some patients can tolerate the food once (e.g., in the OFC), but they develop symptoms with re-exposure at home, as previously reported [62,78].

The introduction of an alternative species of fish will help to avoid extended dietary restrictions, at least until acquisition of tolerance to the offending fish has been achieved. In addition, it appears that consumption of another type of fish could promote the acquisition of tolerance to the original offender, which otherwise, during the natural course, would develop later. After conducting oral immunotherapy with hake, a child with IgE-mediated fish allergy may be able to tolerate other types of fish [79]. Oral desensitization in egg-induced FPIES has been reported recently, but no data are yet available on the active induction of tolerance in fish-FPIES [80].

**Figure 1:**
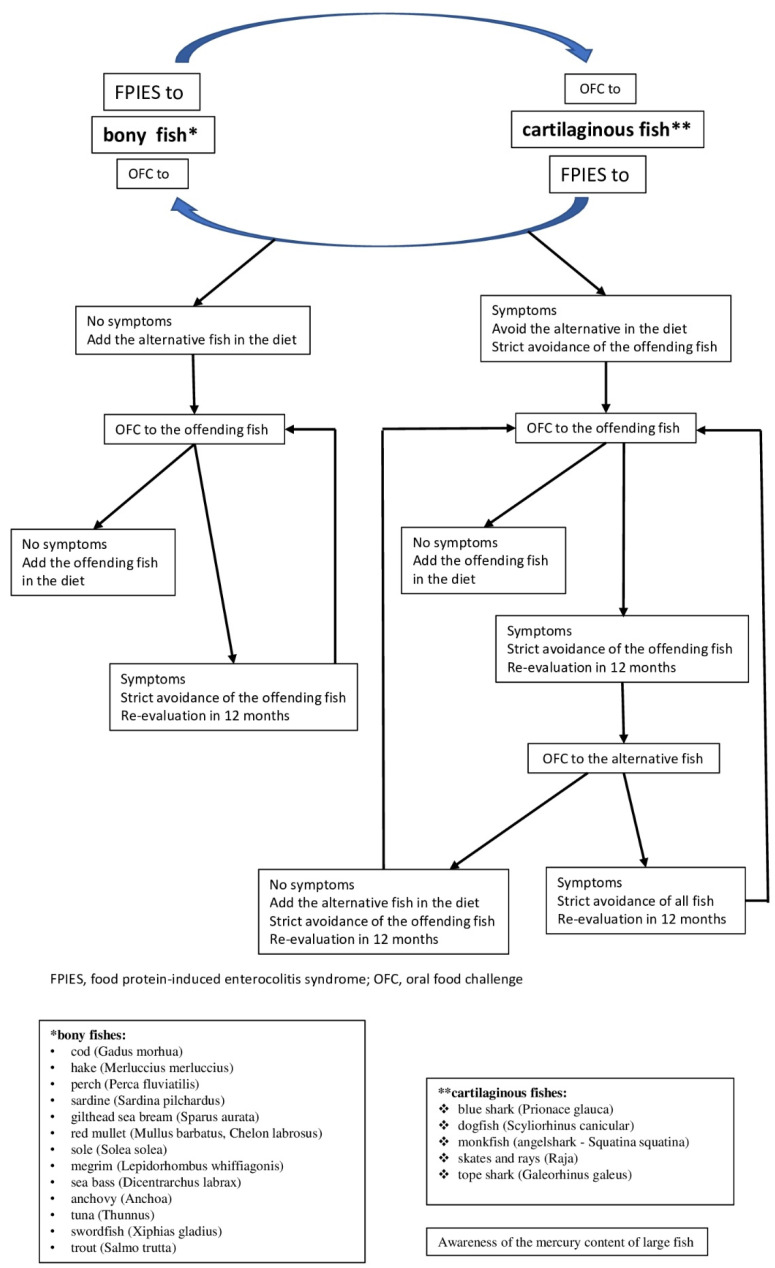
Algorithm for the management of food protein-induced enterocolitis syndrome (FPIES) caused by fish. * bony fishes: cod (*Gadus morhua*), hake (*Merluccius merluccius*), perch (*Perca fluviatilis*), sardine (*Sardina pilchardus*), gilthead sea bream (*Sparus aurata*), red mullet (*Mullus barbatus*, *Chelon labrosus*), sole (*Solea solea*), megrim (*Lepidorhombus whiffiagonis*), sea bass (*Dicentrarchus labrax*), anchovy (*Anchoa*), tuna (*Thunnus*), swordfish (*Xiphias gladius*), trout (*Salmo trutta*); ** cartilaginous fishes: blue shark (*Prionace glauca*), dogfish (*Scyliorhinus canicular*), monkfish (angelshark—*Squatina squatina*), skates and rays (*Raja*), tope shark (*Galeorhinus galeus*) (Information on fish species identification from www.fishbase.org (accessed on 1 November 2021) [28]).

Therefore, we consider that following diagnosis of FPIES due to a specific type of fish, an OFC should be conducted with an alternative fish species. As a result of the quantitative and qualitative differences in protein content between bony and cartilaginous fish, and based on our clinical experience, we believe that it is worthwhile to include in the guidelines for OFC the option to conduct a challenge test to cartilaginous fish, in the case of FPIES to bony fish, and vice versa. Based on the clinical experience of each physician and the familial dietary habits, different other fish species can be used as alternative solutions to a certain offending fish species. We propose an algorithm for the management of FPIES caused by a certain fish, including OFC with alternative fish species (Figure 1).

The introduction of other fish species in the diet of children with FPIES caused by a certain species can contribute to the faster acquisition of tolerance to the incriminated species, which is a phenomenon already reported in IgE allergy to fish [79]. Even if it remains to be proven, we believe that this algorithm will be useful to clinicians in managing FPIES and that it will provide the basis for further studies.

## 5. Conclusions

Children with fish-FPIES are generally recommended fish avoidance regardless of species. No laboratory tests are available to investigate tolerance, and OFC is currently the gold standard to demonstrate tolerance. Testing with fish species other than the type incriminated might identify one or more alternatives that can be tolerated. As a result of their different protein content and/or composition, a logical alternative from the point of view of the allergist would be to conduct an OFC with a cartilaginous fish in the case of allergy to a bony fish and vice versa. Specific consideration should be given in the maximum weekly mercury intake contained in some large species of fish, as imposed by the EFSA. Regular consumption of alternative fish can lead to the acquisition of tolerance for the offending fish.

## Figures and Tables

**Table 1:** Fish species implicated in the presentation of FPIES in children.

Fish Species	Number of Cases (%)	Tolerance to Other Species	Country, City	Publication
hake, whiting, sole, perch, anchovy, monkfish	16	Unspecified	Spain, Alicante	[6]
hake	3 (37.5%)	Unspecified	Spain, Madrid	[7]
hake, sole, megrim, cod, canned tuna, sardine, swordfish	80	canned tuna and swordfish	Spain, Madrid	[16]
cod, perch, sardine, tope, sea bream	56 (56%)	5 subjects tolerated a type of fish other than the culprit species	Greece, Athens	[10]
cod, tope shark, tuna	25 (34.7%)	Unspecified	Greece, Athens	[9]
unspecified	42 (53.8%)	Unspecified	Greece, multicenter	[8]
unspecified fish, white fish, tuna, salmon	12 (5%)	Unspecified	Australia (multicenter)	[52]
hake (14) monkfish (6), sole and megrim (4)	17 (80%)	Unspecified	Spain, La Coruna	[4]
hake, sole, cork float	14	Unspecified	Spain, Madrid	[1]
sole, cod, sea bass, gilthead, anchovy	70	cod, salmon, swordfish, bass, red mullet, anchovy, canned tuna, gilthead, trout	Italy, multicenter	[3]
hake (19), sole (9), monkfish (7), canned tuna (4), salmon (2), swordfish (1), fresh tuna (1), dogfish (1)	44 (54.3%)	Other fish species were not tested.	Spain, Barcelona	[5]
unspecified fish	5 (3.12%)		US, New York	[73]
cod, sole, sea bream, salmon, trout	8 (12%)	three were tolerant to different fish types (salmon + swordfish, cod + tuna, sea bream + cod + perch)	Italy multicenter	[2]
unspecified	102 (57%)	41% reacted to more than one fish species and 78/102 (76%) were avoiding all fish.	Spain, Italy, 12 centers	[11]
unspecified	28 (25%)	Unspecified	Sweden, multicenter	[74]
unspecified	19 (14%)	Unspecified	UK and Ireland	[75]
unspecified	39 (32.5%)	Unspecified	Spain, multicenter	[76]
unspecified	19 (11%)	Unspecified	Australia	[77]
